# Response to Letter to the Editor From Virú-Loza and Chávez-Nomberto: “Safety and Efficacy of Pediatric Growth Hormone Therapy: Results From the Full KIGS Cohort”

**DOI:** 10.1210/clinem/dgad055

**Published:** 2023-01-31

**Authors:** Mohamad Maghnie, Michael B Ranke, Mitchell E Geffner, Elpis Vlachopapadopoulou, Lourdes Ibáñez, Martin Carlsson, Wayne Cutfield, Raoul Rooman, Roy Gomez, Michael P Wajnrajch, Agnès Linglart, Renata Stawerska, Peter E Clayton, Feyza Darendeliler, Anita C S Hokken-Koelega, Reiko Horikawa, Toshiaki Tanaka, Helmuth-Günther Dörr, Kerstin Albertsson-Wikland, Michel Polak, Adda Grimberg

**Affiliations:** Department of Pediatrics, IRCCS Giannina Gaslini, Genova 16124, Italy; Department of Neuroscience, Rehabilitation, Ophthalmology, Genetics, Maternal and Child Health - DINOGMI, University of Genova, Genova 16124, Italy; Department of Pediatric Endocrinology, University Children´s Hospital, Tübingen 72076, Germany; The Saban Research Institute, Children's Hospital Los Angeles, Los Angeles, CA 90027, USA; Department of Endocrinology, Growth and Development, Aglaia Kyriakou Children's Hospital, Athens 11527, Greece; Endocrinology, Pediatric Research Institute Sant Joan de Déu, Barcelona 08950, Spain; Centro de Investigación Biomédica en Red de Diabetes y Enfermedades Metabólicas Asociadas (CIBERDEM), Instituto de Salud Carlos III, Madrid 28029, Spain; Rare Disease, Biopharmaceuticals, Pfizer, New York, NY 10017, USA; Liggins Institute, University of Auckland, Auckland 1142, New Zealand; PendoCon, Putte 2580, Belgium; European Medical Affairs, Pfizer, Brussels 1070, Belgium; Rare Disease, Biopharmaceuticals, Pfizer, New York, NY 10017, USA; Department of Pediatrics, New York University Langone Medical Center, New York, NY 10016, USA; Department of Pediatric Endocrinology and Diabetology for Children, AP-HP, Bicêtre Paris Saclay, Le Kremlin Bicêtre 94270, France; APHP, Reference Center for Rare Disorders of the Calcium and Phosphate Metabolism, Filière OSCAR and Plateforme d’Expertise Maladies Rares Paris-Sud, Bicêtre Paris Saclay Hospital, Le Kremlin Bicêtre 94270, France; Department of Endocrinology and Metabolic Diseases, Polish Mother's Memorial Hospital-Research Institute, Lodz 93-338, Poland; Department of Pediatric Endocrinology, Medical University of Lodz, Lodz 93-338, Poland; Developmental Biology and Medicine, Faculty of Biology Medicine and Health, Manchester NIHR Academic Health Science Centre, University of Manchester, Manchester M13 9PL, UK; Istanbul University, Istanbul Faculty of Medicine, Pediatric Endocrinology Unit, İstanbul 34452, Turkey; Pediatrics, Subdivision of Endocrinology, Erasmus University Medical Center, Rotterdam 3015 GD, the Netherlands; Division of Endocrinology and Metabolism, National Center for Child Health and Development, Tokyo 157-8535, Japan; Tanaka Growth Clinic, Tokyo 158-0097, Japan; Division of Pediatric Endocrinology, Department of Pediatrics and Adolescent Medicine, Friedrich-Alexander University of Erlangen Nürnberg, Erlangen 91054, Germany; Department of Physiology/Endocrinology, Institute of Neuroscience and Physiology, Sahlgrenska Academy, University of Gothenburg, Gothenburg 405 30, Sweden; Université de Paris Cité; Hôpital Universitaire Necker Enfants Malades, Paris 75015, France; Division of Pediatric Endocrinology and Diabetes, Children's Hospital of Philadelphia, Philadelphia, PA 19104, USA

To the Editor,

We thank Virú-Loza and Chávez-Nomberto for highlighting some of the limitations and concerns raised in the discussion section of our paper, “Safety and Efficacy of Pediatric Growth Hormone Therapy: Results From the Full KIGS Cohort” (1), and agree with their conclusion “that longer follow-up and adequate comparisons are needed to have better information on whether or not there is a higher incidence of some types of cancer or higher-risk subgroups” (2). Our paper was never intended as the final imprimatur of the safety of pediatric growth hormone (GH) therapy; it could not be. In the face of worrisome indirect evidence, challenges and limitations in the direct evidence of long-term (especially into adulthood) safety, as well as changes in both GH treatment practices and its recipients over time, continued scrutiny of GH safety is needed (3). Rather, our paper was intended to leverage real-world clinical data on more than 80 000 children treated with recombinant human GH in more than 50 countries to provide, as described by Abucham and Boguszewski, “a unique and valuable source of good-quality documentation of diverse growth disorders and their treatment outcomes” (4).

## Disclosures

M.M. has received research support from Merck Serono and Pfizer, and has consulted for Ascendis, BioMarin, Ferring, Merck Serono, Novo Nordisk, Pfizer, and Sandoz. M.B.R. has received speaker fees from Mediagnost, Merck, Pfizer, and Sandoz. M.E.G. has a research contract from Novo Nordisk; serves as a consultant for and/or on advisory boards for Adrenas, Eton Pharmaceuticals, Neurocrine Biosciences, Novo Nordisk, and Pfizer; is a member of a data safety monitoring board for Aeterna Zentaris and Ascendis; and receives royalties from McGraw-Hill and UpToDate. E.V. has received research support from Ascendis, OPKO, and Pfizer. W.C. has received research support from Pfizer. R.R. is a past member of the KIGS Strategic Advisory Board and serves as a consultant for Pfizer. A.L. has received speaker fees from Alexion, Kyowa Kirin, Novo Nordisk, Pfizer, and Sandoz. A.C.S.H.-K. is a past member of the KIGS Strategic Advisory Board; recipient of investigator-initiated independent research grants from Novo Nordisk and Pfizer; and has received lecture fees from Merck-Serono, Novo Nordisk, and Pfizer. T.T. has consulted for JCR Pharmaceuticals. H.G.D. has received honoraria for lectures from Ferring, Ipsen, Novo Nordisk, and Pfizer. M.P. is on the advisory board for IPSEN Increlex Registry, Novo Nordisk, and Pfizer France; has received grants from Ipsen, Merck, Novo Nordisk, Pfizer, Sandoz, and Sanofi; and has French institutional public grants from ANR PHRC. A.G. has consulted for Pfizer and received an investigator-initiated independent research grant from Pfizer. R.S. has received independent research support from OPKO, Pfizer, and Sandoz. M.C., R.G., and M.P.W. are employees and stockholders/stock grant holders of Pfizer. L.I., P.E.C., F.D., R.H., and K.A.-W. have nothing to disclose.

## Abbreviations

GH: growth hormone
